# EMAP/EMAPA ontology of mouse developmental anatomy: 2013 update

**DOI:** 10.1186/2041-1480-4-15

**Published:** 2013-08-26

**Authors:** Terry F Hayamizu, Michael N Wicks, Duncan R Davidson, Albert Burger, Martin Ringwald, Richard A Baldock

**Affiliations:** 1 The Jackson Laboratory, Bar Harbor, USA; 2 MRC Human Genetics Unit, Institute of Genetics and Molecular Medicine, Edinburgh, UK; 3 Department of Computer Science, Heriot-Watt University, Edinburgh, UK

**Keywords:** Mouse development, Anatomy ontology, Developmental anatomy, OBO format

## Abstract

**Background:**

The Edinburgh Mouse Atlas Project (EMAP) ontology of mouse developmental anatomy provides a standard nomenclature for describing normal and mutant mouse embryo anatomy. The ontology forms the core of the EMAP atlas and is used for annotating gene expression data by the mouse Gene Expression Database (GXD), Edinburgh Mouse Atlas of Gene Expression (EMAGE) and other database resources.

**Findings:**

The original EMAP ontology listed anatomical entities for each developmental stage separately, presented as uniparental graphs organized as a strict partonomy. An "abstract" (i.e. non-stage-specific) representation of mouse developmental anatomy has since been developed. In this version (EMAPA) all instances for a given anatomical entity are presented as a single term, together with the first and last stage at which it is considered to be present. Timed-component anatomies are now derived using staging information in the "primary" non-timed version. Anatomical entities are presented as a directed acyclic graph enabling multiple parental relationships. Subsumption classification as well as partonomic and other types of relationships can now be represented. Most concept names are unique, with compound names constructed using standardized nomenclature conventions, and alternative names associated as synonyms.

**Conclusions:**

The ontology has been extended and refined in a collaborative effort between EMAP and GXD, with additional input from others. Efforts are also underway to improve the revision process with regards to updating and editorial control. The revised EMAPA ontology is freely available from the OBO Foundry resource, with descriptive information and other documentation presented in associated Wiki pages (http://www.obofoundry.org/wiki/index.php/EMAPA:Main_Page).

## Findings

### EMAP ontology

The ontology of mouse developmental anatomy was originally developed by Jonathan Bard and his colleagues as part of the Edinburgh Mouse Atlas Project (EMAP; www.emouseatlas.org) in order to provide a structured controlled vocabulary of stage-specific anatomical structures for the developing laboratory mouse [1]. In order to construct the original dictionary of anatomy terms, histologically distinguishable anatomical entities were identified and organized as simple, strictly uniparental hierarchies (trees). Initial selection of terms was based on the tissue index for The Atlas of Mouse Development [2]. Subsequently, the list of anatomical terms was substantially extended. Term names were assigned based on what was considered to be most generally accepted names, with synonyms included as appropriate. Individual term labels were not necessarily unique, but each component could be unambiguously specified by its "full name", which included its ordered hierarchical path, as well as by a unique numerical identifier (i.e. EMAP ID). For example, the term for "epithelium" associated with id EMAP:969 could be specified by its full hierarchical path, i.e. TS14/mouse/organ system/visceral organ/alimentary system/gut/midgut/epithelium.

The original hierarchy only utilized "part-of" relationships, based primarily on structural subdivisions. The intent was to describe the whole embryo as a tree of anatomical structures successively divided into non-overlapping named parts. Sets of anatomical terms for each standardised developmental stage (Theiler Stage, TS) [3], were presented as separate hierarchical trees. For example, at TS20, the mouse embryo has parts (e.g. head, limb, trunk and tail) which are progressively subdivided, e.g. limb > forelimb > handplate > digit 1 > mesenchyme.

**Figure 1:**
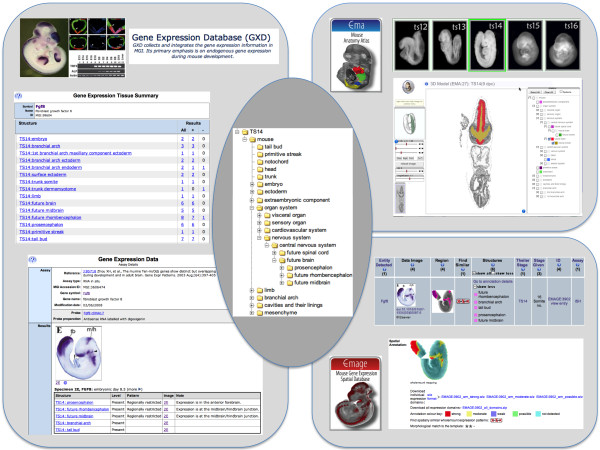
**EMAP ontology: Mouse developmental anatomy and gene expression data.** The original EMAP ontology has been and is still being used for standardised description of anatomical entities by the e-Mouse Atlas (EMA), an anatomical atlas of mouse embryo development, and for annotation of gene expression data by the Gene Expression Database for Mouse Development (GXD) and the e-Mouse Atlas of Gene Expression (EMAGE).

EMAP terms, organized within trees for each Theiler stage, have been adopted for the annotation of expression data by the Gene Expression Database for Mouse Development (GXD; http://www.informatics.jax.org/expression.shtml), part of the Mouse Genome Informatics (MGI) resource at The Jackson Laboratory, and the Edinburgh Mouse Atlas of Gene Expression (EMAGE; http://www.emouseatlas.org/emage). Figure 1 illustrates the role of EMAP as the means of integration between GXD and EMAGE. Other database resources currently utilizing EMAP ontology terms include EurExpress (http://www.eurexpress.org) and the GenitoUrinary Molecular Anatomy Project (GUDMAP; http://www.gudmap.org). In addition, the EMAP ontology forms the core of the EMAP anatomical atlas (http://www.emouseatlas.org/emap) and will be an important element of the online version of the Atlas of Mouse Development [2]. Finally, EMAP terms as well as the hierarchical organization of the ontology were used as a framework for construction of an anatomy ontology for the postnatal mouse by GXD [4]. This has enabled consistency of nomenclature and will facilitate future integration of these ontologies.

### The "abstract mouse"

From the onset, the design of the EMAP database identified each Theiler stage-dependent term as a "timed-component" with a hidden "abstract mouse" [1] comprised of a set of stage-independent terms with partonomic relationships. The abstract mouse anatomy ontology was algorithmically derived from the existing stage-dependent anatomy hierarchies by forming the union of all stage-dependent graphs where nodes represent anatomical structures and edges represent part-of links [5]. Nodes in the abstract mouse graph represent anatomical structures that exist during some time of embryo development and broadly correspond to so-called "material continuants" [6] (http://code.google.com/p/obo-relations/). Originally invented as a schema design for the object-oriented database system used to store the anatomy, the idea of an abstract mouse has subsequently proved useful at a conceptual level. The non-timed version of the mouse developmental anatomy ontology has previously been made available on a limited basis, with unique identifiers included as persistent, trackable IDs.

### Updates to EMAPA

The stage-dependent EMAP hierarchies have provided a valuable basis for data annotation and integration, but various inherent limitations have been encountered. Early on, it became apparent that the ability to provide alternate representations of the anatomy would be required, with different hierarchial views enabling classification and other types of relationships. Also problematic were the inherent constraints in cases where the embryonic age or stage was poorly or not specified. Another issue was the fact that term labels, such as "epithelium" were originally not necessarily unique, nor specific. It was clear that the ontology would benefit from a series of modifications. Pursuant to these goals, the "abstract" version of the mouse developmental anatomy has since been further developed.

**Figure 2:**
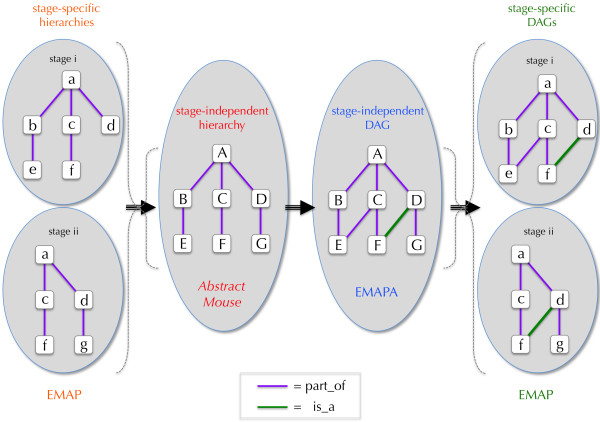
**EMAP and EMAPA ontologies provide stage-specific and stage-independent representations of mouse embryo anatomy.** Originally constructed as uniparental partonomic hierarchies with anatomic entities for each Theiler stage of embryonic development, the anatomy ontology for mouse development has been revised and is now comprised of directed acyclic graphs (DAGs) with both stage-independent and stage-specific representation for mouse developmental anatomy.

The uniparental hierarchy was converted to a directed acyclic graph (DAG) enabling multiple parental relationships (see Figure 2). This allowed the representation of anatomical concepts that were otherwise not possible. For example, "brain" can be represented as a part of "head" as well as a part of "central nervous system". The DAG format also supported the inclusion of other types of relationships as well as partonomic ones. Subsumption classification and other relationship types can now be represented. In the revised EMAPA representation, all instances for a given anatomical entity are presented as a single term, together with the first and last stage at which the entity is considered to be present in the developing embryo. Stage-specific EMAP anatomy hierarchies are now derived using staging information associated with terms in the "primary" non-timed EMAPA version. The ontology has also been transformed into a more supportable format based on openly available relational database technology, coupled with a standard input/output format developed by the Open Biological Ontologies (OBO) consortium. These changes have and will continue to facilitate further development of the ontology.

In extensions to the EMAPA ontology, "is-a" relations have been introduced (Figure 2) in situations where this relationship was determined to be more appropriate than "part-of" (e.g., nervous system is-a organ system). The use of "is-a" relations has also been used in extensions to the ontology to facilitate data annotation and to support subsumption classification of anatomical entities in the ontology. In general, modeling of hierarchial relationships has followed the conventions identified by GXD in developing the ontology for postnatal mouse anatomy (MA) [4]. These conventions also parallel those being adopted for anatomy ontologies by other model organism databases, as well as scientific community-wide efforts developing multispecies ontologies (see below).

In the original version of the EMAP ontology, individual term labels were not necessarily unique, often requiring knowledge of the hierarchical path for disambiguation. Because it was impractical to display the full path names in user interfaces, shortened "print names" have been implemented. For example, to represent expression results for the above mentioned anatomical structure EMAP:969 in GXD, the print name "TS14;midgut epithelium" was displayed rather than the the full path name or the ambiguous term label "epithelium". Term identification based on parental hierarchy was further complicated by the introduction of multiple parentage. Consequently, in an effort to provide unique names for all terms, every term name in the ontology was evaluated for uniqueness. In numerous cases, modified compound names were constructed for many terms using standardized nomenclature conventions [4]. Alternative names will continue to be added as synonyms. The evaluation of this and other nomenclature considerations will remain as part of the editorial process.

Furthermore, the ontology has been substantially extended and refined in collaborative efforts between EMAP and GXD. The original EMAP ontology contained more than 14,200 stage-specific terms for anatomical entities in the mouse embryo, corresponding to about 3,400 "abstract" anatomy terms. Since then, terms have been added, predominantly in response to the requirements of substantial amounts of gene expression data curation by both GXD [7] and EMAGE [8]. In addition, urinary and reproductive systems have been extensively extended and refined by curators from GUDMAP [9]. Based on the information contained in the EMAPA file, stage-specific terms with associated EMAP identifiers have been instantiated. The resulting set of EMAP terms and identifiers includes and is consistent with previous versions of the mouse developmental anatomy. Currently, the EMAPA ontology includes 5,590 anatomy terms, corresponding to over 35,000 stage-specific EMAP terms.

The anatomical ontology for the developing mouse will continue to be expanded and refined based on additional resources, as well as the needs of the scientific community. The revised EMAPA ontology has been made freely available as a text file in OBO format via the OBO Foundry resource (http://www.obofoundry.org). Obo-formatted files containing EMAP ontology hierarchies for each of the Theiler stages for mouse development, presented as separate DAGs, will also be available. In addition, in order to facilitate interoperability of resources using different sets of mouse anatomy terms, a mapping file has been created in which all corresponding EMAP and EMAPA terms have been specified. Descriptive information and other documentation relevant to these files is provided in associated Wiki pages. Stage-specific EMAP and "abstract" EMAPA ontologies can also be accessed at the EMAP site (http://www.emouseatlas.org/emap/ema/DAOAnatomyJSP/abstract.html) using a browser which enables searching for terms directly as well as "browsing" through the respective hierarchies.

### Future directions

The EMAPA ontology, along with instantiated stage-specific EMAP components, will continue to be expanded and refined according to the requirements of data curation and input from the scientific community at large. Optimally, as in the case of the GUDMAP contributions, this will include editing of specific areas of the ontology with domain expert involvement. Efforts to improve the revision process with regards to updating and editorial control are also underway. Plans are being developed in order to facilitate term requests, and to enable appropriate editorial tracking and version control. Future development of the EMAPA ontology itself will also involve extension and refinement of relationships between concepts, including further development of the subsumption classification hierarchy, as well as introduction of other types of relationships. Particularly, the "develops-from" relationships will be included to support the analysis of differentiation pathways in databases that deal with expression, phenotypic, and disease-related information. Another goal is the inclusion of a set of textual definitions, computable logical definitions that can be used by automated reasoners, and other forms of metadata. Further efforts are underway towards adhering to basic ontological principles such as those set forth by the OBO Foundry [10].

The new EMAPA ontology will be used by GXD, EMAGE, and EMAP, as well as by other resources that have employed previous versions of the ontology to describe gene expression patterns and other biological data pertinent to mouse anatomy. These include Gene Ontology (GO) [11] for annotation of mouse gene products, as well as several efforts utilizing the entity-quality (EQ) approach [12] to describe data annotated using the Mammalian Phenotype Ontology (MP) [13]. EMAPA terms and identifiers are also included in bridging extensions to the Uberon multispecies anatomy ontology [14], which will further serve to facilitate integration of mouse developmental data within the broader scientific domain. New research has also been initiated to study how an ontology such as EMAP can be used to integrate experimental data from model organisms, such as the EMAGE database, with a computational framework of human physiological modelling for eHealth purposes (part of the Virtual Physiological Human programme), though this work is still very preliminary [15].

### Conclusion

Here we have presented the recently updated and extended EMAP ontology of mouse developmental anatomy. The ontology has been in active use for many years in GXD and EMAGE for annotation of gene-expression data and as part of the Edinburgh Mouse Atlas model framework. Since the original development of the ontology, the modelling emphasis has shifted from a series of time-dependent ontologies to a single "abstract" time-independent ontology (EMAPA), where the former can now be automatically derived from the latter. The ontology is available from the OBO Foundry web-site and is under continuous revision to include new terms and relationships. In particular the ontology will be updated to ensure a full class hierarchy for each tissue term and extension of the lineage information encoded via the "develops-from" relationship. This extension will enable automated consistency checking and validation in addition to the semantic checking provided by the editorial review group.

## Abbreviations

DAG: Directed acyclic graph; EMAGE: Edinburgh Mouse Atlas of Gene Expression; EMAP: Edinburgh Mouse Atlas Project; GXD: Gene Expression Database for mouse development at MGI; GUDMAP: GenitoUrinary Molecular Anatomy Project; MGI: Mouse Genome Informatics at The Jackson Laboratory, USA; OBO: Open Biological Ontologies.

## Competing interests

The authors declare that they have no competing interests.

## Authors’ contributions

TFH undertook the primary effort to update and extend the EMAPA ontology to its present form and wrote the first draft of this manuscript. MNW has developed the underlying database and associated code to provide validation of the ontology and production of the new version with consistent UIDs maintaining persistence as required. DRD, AB, MR and RAB have contributed in the overall ontology design and provided expert input either in terms of developmental anatomy or ontology formalisation aspects or both. All authors read and approved the final manuscript.
